# First-trimester SPISE mediates the association between early-pregnancy body fat percentage change and gestational diabetes mellitus: an exploratory secondary analysis of a prospective cohort

**DOI:** 10.3389/fendo.2026.1931823

**Published:** 2026-09-09

**Authors:** Xuran Zhang, Meiyuan Guo, Yingjie Chen

**Affiliations:** 1 Department of Breast Surgery, Shanxi Provincial People’s Hospital, Taiyuan, Shanxi, China; 2 Department of Obstetrics, Shishi Municipal Hospital, Quanzhou, Fujian, China; 3 Department of Emergency, Shishi Municipal Hospital, Quanzhou, Fujian, China

**Keywords:** body fat percentage, early pregnancy, gestational diabetes mellitus, insulin sensitivity, mediation analysis, secondary analysis, SPISE

## Abstract

**Background:**

Gestational diabetes mellitus (GDM) is closely related to maternal adiposity and insulin resistance. The rate of body fat percentage (BFP) change in early pregnancy may describe estimated BFP gain before routine glucose screening. The Single-Point Insulin Sensitivity Estimator (SPISE) offers a fasting estimate of insulin sensitivity without insulin testing. It is still uncertain whether SPISE explains the link between early BFP change and later GDM.

**Methods:**

We analyzed public, de-identified data from a prospective Korean cohort of singleton pregnancies. BFP was estimated from age and body mass index. Early-pregnancy estimated BFP change rate was defined as BFP at 10–14 weeks minus pre-pregnancy BFP, divided by gestational week at assessment, and rescaled to per mille units. SPISE was calculated from fasting triglycerides, high-density lipoprotein cholesterol, and body mass index. GDM was assessed at 24–28 weeks with a two-step diagnostic approach. The analyses used restricted cubic splines, segmented regression, mediation models, and propensity score matching.

**Results:**

Among 586 women in the analytic cohort, 37 (6.3%) developed GDM. Higher estimated BFP change rate was linked to greater GDM risk (P overall = 0.006; P non-linearity = 0.140). SPISE showed a non-linear inverse association with GDM (P overall < 0.001; *P* non-linearity = 0.003), with an estimated threshold of 7.089. Below this threshold, each one-unit increase in SPISE was linked to lower GDM odds (OR 0.127, 95% CI 0.049-0.328; P < 0.001). Above the threshold, the association was weaker and not significant. estimated BFP change rate was also inversely associated with SPISE. In mediation analysis, the model-based estimated proportion mediated was 43.65%. After propensity score matching, available-case outcomes showed more GDM events in the low-SPISE group than in the high-SPISE group (14.5% vs 0.0%; P = 0.003).

**Conclusions:**

Faster estimated BFP change in early pregnancy was linked to higher later GDM risk, and lower SPISE explained part of this relationship in statistical mediation models. The proposed SPISE threshold and mediation pattern need validation in independent cohorts before clinical use.

## 1 Introduction

Gestational diabetes mellitus (GDM) is a common metabolic complication of pregnancy, and its effects extend beyond delivery. It increases obstetric and neonatal risk and is linked to later cardiometabolic disease in mothers and offspring (1–5). Routine screening is usually performed at 24–28 gestational weeks, so earlier markers could help identify women who need closer follow-up or preventive support (6, 7).

Maternal adiposity is central to GDM biology. Pre-pregnancy obesity, altered lipid metabolism, and early-gestation insulin resistance have been linked to later glucose intolerance (8–11). Body mass index (BMI) is convenient, but it does not distinguish fat mass from lean mass and is affected by age, sex, and ethnicity (12, 13). BFP estimated from BMI and age is a practical adiposity measure for cohort studies (12). A dynamic measure, such as the estimated BFP change rate from before pregnancy to the first trimester, may capture early metabolic adaptation better than a single body-size value.

SPISE is a previously developed and validated surrogate index of insulin sensitivity, not a prediction model newly trained in the present cohort (14). It is calculated from fasting triglycerides, HDL-C, and concurrent BMI; higher values indicate better insulin sensitivity. In the present analysis, the BMI term was BMI at 10–14 weeks. Prepregnancy BMI and age were used separately to estimate BFP change and for covariate adjustment and were not components of SPISE. Because the SPISE inputs are often available in prenatal cohorts, the index can support population-level research when direct insulin measurements are unavailable. Its association with later GDM and its possible statistical role between adiposity change and GDM remain uncertain.

We used data from a prospective Korean pregnancy cohort originally designed to study fatty liver in pregnancy (15). We examined dose-response patterns among early-pregnancy BFP change, SPISE, and GDM; tested for a SPISE threshold in the SPISE-GDM association; and evaluated whether SPISE mediated the relationship between BFP change and GDM. This exploratory analysis was designed to generate hypotheses for external testing.

## 2 Methods

### 2.1 Study design and data source

This study was an exploratory secondary analysis of de-identified public data from the Fatty Liver in Pregnancy prospective cohort registry (ClinicalTrials.gov identifier NCT02276144), conducted in South Korea from November 2014 to July 2016 (15). The original cohort enrolled singleton pregnant women receiving prenatal care at Seoul Metropolitan Government Seoul National University Boramae Medical Center and Incheon Seoul Women’s Hospital. The dataset and primary report were published in PLoS One under an open license (15). This manuscript follows the STROBE reporting framework for observational studies (16).

### 2.2 Ethics statement

The source cohort was approved by the Public Institutional Review Board of the Korean Ministry of Health and Welfare and the Institutional Review Board of Seoul Metropolitan Government Seoul National University Boramae Medical Center. All participants provided written informed consent before enrollment. This secondary analysis used de-identified data and followed the Declaration of Helsinki (17).

### 2.3 Study population

**Figure 1:**
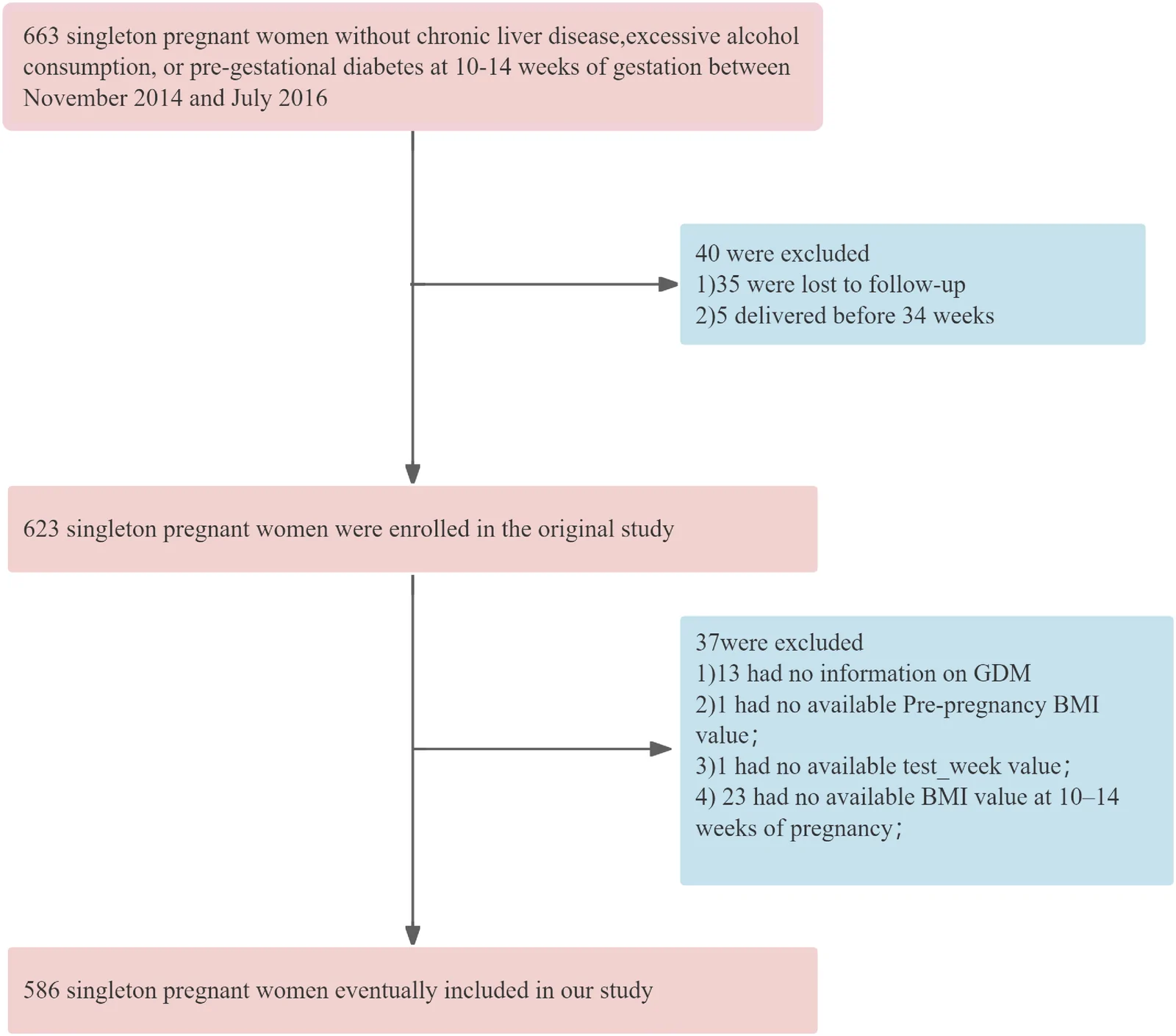
Flow diagram showing participant selection. The source cohort included 663 singleton pregnant women assessed at 10–14 gestational weeks. Forty women were excluded because of loss to follow-up or delivery before 34 weeks, leaving 623 participants. A further 37 participants were excluded because of missing GDM status, prepregnancy BMI, gestational week at assessment, or variables required to reconstruct first-trimester BMI. The final complete-case cohort comprised 586 women, including 37 who subsequently developed GDM.

The source cohort enrolled 663 women with singleton pregnancies who were assessed at 10–14 gestational weeks. Forty women were excluded because they were lost to follow-up or delivered before 34 weeks. Of the remaining 623 participants, 37 were excluded from the complete-case analysis because GDM status, prepregnancy BMI, gestational age at assessment, or variables needed to reconstruct first-trimester BMI were missing. The final analytic cohort included 586 women (**Figure 1**).

### 2.4 Data collection

BFP was estimated using the adult Deurenberg equation: BFP (%) = 1.20 x BMI + 0.23 x age - 10.8 x sex - 5.4 (12). All participants were women, so sex was coded as 0, yielding BFP (%) = 1.20 x BMI + 0.23 x age - 5.4. Pre-pregnancy BFP was calculated from pre-pregnancy BMI and age.

BMI at 10–14 weeks was reconstructed from the hepatic steatosis index (HSI). HSI is defined as 8 x (ALT/AST) + BMI + 2 for female sex, with an additional +2 for diabetes mellitus (18). Because participants were female and did not have pregestational diabetes, BMI at 10–14 weeks was calculated as HSI - 8 x (ALT/AST) - 2. BFP at 10–14 weeks was then estimated with the same BFP equation. Early-pregnancy estimated BFP change rate was calculated as (BFP at 10–14 weeks - pre-pregnancy BFP) divided by gestational week at testing. This weekly percentage-point value was multiplied by 10 and reported in per mille units.

SPISE was calculated as 600 x HDL-C^0.185^/(triglycerides^0.2^ x BMI^1.338^), using fasting HDL-C and triglyceride concentrations in mg/dL and BMI in kg/m² (14). Higher SPISE values indicate greater insulin sensitivity. The BMI term was BMI at 10–14 weeks; prepregnancy BMI, age, and other variables were not components of SPISE. Thus, SPISE was an existing index applied to this cohort rather than a newly developed prediction model.

### 2.5 Outcome: GDM

At 24–28 gestational weeks, participants underwent GDM screening with the two-step method recommended by the American College of Obstetricians and Gynecologists. The 50 g oral glucose challenge test measured serum glucose 1 hour after glucose intake. GDM was diagnosed when at least two Carpenter-Coustan thresholds were met or exceeded: fasting ≥ 5.3 mmol/L, 1 hour ≥ 10.0 mmol/L, 2 hours ≥ 8.6 mmol/L, or 3 hours ≥ 7.8 mmol/L (6, 7, 19).

### 2.6 Covariates

Covariates were selected according to clinical usefulness and data availability. They included maternal age, parity, pre-pregnancy BMI, and gestational week at blood sampling when applicable. Subgroup definitions used conventional cut-points for advanced maternal age (35 years) and BMI-related risk in Asian populations (23 kg/m²) (13, 20).

### 2.7 Statistical analysis

Primary analyses used the complete-case cohort, with participant numbers reported for each model. Analyses were conducted in R Statistical Software (v4.2.2; R Foundation for Statistical Computing) and the Free Statistics analysis platform (v2.5; Beijing, China). FreeStatistics uses R as its statistical engine and provides graphical interfaces for common analyses and visualization. A two-sided P value <0.05 was considered statistically significant.

SPISE was approximately normally distributed, as assessed by the Kolmogorov-Smirnov test. Normally distributed continuous variables are presented as mean ± SD, and skewed variables as median (interquartile range). Categorical variables are reported as n (%).

Dose-response associations were examined with generalized additive models and restricted cubic splines for three relationships: estimated BFP change rate with GDM, SPISE with GDM, and estimated BFP change rate with SPISE. For all RCS analyses, four knots were placed at the 5th, 35th, 65th, and 95th percentiles of the exposure distribution, and the overall and nonlinear associations were assessed using Wald chi-square tests (21, 22). The estimated BFP change rate-GDM logistic RCS model was adjusted for maternal age, parity, and prepregnancy BMI. The SPISE-GDM logistic RCS model was additionally adjusted for gestational week at testing. These covariates were selected *a priori* on the basis of previous literature and clinical considerations rather than univariable statistical significance. When non-linearity was detected, segmented regression estimated the breakpoint and piecewise effects (23). Segmented multivariable logistic regression reported adjusted odds ratios (ORs) and 95% confidence intervals (CIs) on each side of the breakpoint. Segmented multivariable linear regression was used when SPISE was the continuous outcome and reported adjusted beta coefficients with 95% CIs. Mediation analysis used bootstrap confidence intervals within the R mediation framework (24–26). estimated BFP change rate was the exposure, GDM was the outcome, and SPISE status at the statistically identified threshold of 7.089 was the mediator. Covariate adjustment was kept consistent with the primary models. All tests were two-sided, and P<0.05 was treated as statistically significant.

The full-cohort estimated BFP change rate-GDM logistic RCS model contained six non-intercept parameters: the four-knot spline term contributed three degrees of freedom, and maternal age, parity, and prepregnancy BMI each contributed one degree of freedom. With 37 GDM events, this corresponded to 6.2 events per parameter (37/6). The full-cohort SPISE-GDM logistic RCS model contained seven non-intercept parameters: three spline degrees of freedom plus four covariate parameters, corresponding to 5.3 events per parameter (37/7). The estimated BFP change rate-SPISE linear RCS model also contained seven non-intercept parameters; an event-based ratio was not applicable because SPISE was continuous. The SPISE-GDM segmented model contained two SPISE slope coefficients and four covariate coefficients; when the data-derived breakpoint was counted as an additional estimated parameter, the analysis involved seven non-intercept parameters, corresponding to 5.3 events per parameter. Model diagnostics were evaluated separately for both logistic RCS models and included comparison of observed and model-predicted outcome counts and variance inflation factors (VIFs), with VIF ≥ 5 considered evidence of substantial multicollinearity (**Supplementary Figures 3**, **S4**). The conventional 10-events-per-variable criterion was treated as a heuristic rather than an absolute threshold because simulation evidence indicates that acceptable performance may occur below 10 events per parameter, particularly when prespecified covariates are retained for confounding control (29, 30).

Several sensitivity analyses were performed to assess the stability of the primary findings and the clinical meaning of the SPISE threshold.

#### 2.7.1 Sensitivity analysis 1: mediation analysis using continuous SPISE

The main mediation model used SPISE dichotomized at the data-derived threshold. To check whether this choice affected the results, we repeated the mediation analysis with SPISE as a continuous mediator. The findings remained similar when SPISE was modeled as a continuous mediator. The R mediation package estimated total, indirect, and direct effects, as well as mediation proportions, using 1, 000 bootstrap replicates with adjustment for age, parity, and prepregnancy BMI.

#### 2.7.2 Sensitivity analysis 2: clinical validation of the SPISE threshold using propensity score matching

Propensity score matching was used to examine whether the statistically identified SPISE threshold corresponded to differences in clinical outcomes. Participants were grouped as SPISE < 7.089 or SPISE ≥ 7.089. Matching was used as a sensitivity analysis to compare outcomes across threshold strata, with covariate balance assessed by standardized mean differences (SMDs) (26, 27). An SMD <0.1 indicated adequate balance. The matched cohort was then used to compare maternal and neonatal outcomes, including GDM, gestational hypertension or preeclampsia, delivery week, preterm birth, cesarean delivery, neonatal birth weight, and large for gestational age (LGA).

#### 2.7.3 Sensitivity analysis 3: ln(HOMA-IR) as an alternative insulin-resistance index

HOMA-IR was calculated as fasting insulin (μU/mL) × fasting glucose (mg/dL)/405 (34). Because untransformed HOMA-IR was right-skewed, the natural logarithm, ln(HOMA-IR), was used for modeling; the distributions of both measures were visualized (**Supplementary Figure 5**). The association between ln(HOMA-IR) and subsequent GDM was examined as an auxiliary restricted cubic spline sensitivity analysis using four knots at the 5th, 35th, 65th, and 95th percentiles and the same prespecified adjustment set as the SPISE-GDM model (maternal age, parity, prepregnancy BMI, and gestational week at testing). The analysis used complete records for HOMA-IR and these covariates. One participant who developed GDM lacked fasting glucose, leaving 585 participants and 36 GDM events. Overall and nonlinear associations were assessed using Wald chi-square tests. Because this analysis was *post hoc* and event-limited, it was considered exploratory. The model contained seven non-intercept parameters, corresponding to 5.1 events per parameter.

## 3 Results

### 3.1 Participant characteristics

**Figure 1** presents the analytic flow. Included and excluded participants had generally comparable baseline characteristics (**Supplementary Table 1**). In the descriptive sample of 586 women, 37 (6.3%) developed GDM. Mean maternal age was 32.1 years, and mean gestational week at early-pregnancy testing was 12.4 weeks. Compared with women without GDM, women who later developed GDM had higher ALT, pre-pregnancy BMI, BMI at 10–14 weeks, pre-pregnancy BFP, and BFP at 10–14 weeks. Unadjusted BFP change measures did not differ significantly between groups (**Table 1**).

**Table 1:** Clinical characteristics of study participants.

Variable	Total (n = 586)	Non_GDM (n = 549)	GDM (n = 37)	*P* value
Parity, n (%)				
Nulliparous	307 (52.4)	288 (52.5)	19 (51.4)	
Parous	279 (47.6)	261 (47.5)	18 (48.6)	0.896
Age, Mean ± SD	32.1 ± 3.8	32.0 ± 3.8	32.5 ± 3.6	0.422
Gestational week at testing, Mean ± SD	12.4 ± 0.5	12.4 ± 0.5	12.4 ± 0.5	0.520
AST, IU/L, Mean ± SD	17.8 ± 8.1	17.7 ± 8.2	19.9 ± 7.3	0.109
ALT, IU/L, Median (IQR)	11.0 (8.0, 15.0)	11.0 (8.0, 14.0)	15.0 (10.0, 25.0)	0.002
Prepregnancy BMI, kg/m2, Mean ± SD	22.0 ± 3.5	21.8 ± 3.2	25.9 ± 5.2	<0.001
BMI at 10–14 weeks, kg/m2, Mean ± SD	22.4 ± 3.5	22.1 ± 3.2	26.7 ± 5.0	<0.001
Prepregnancy BFP, %, Mean ± SD	28.4 ± 4.3	28.1 ± 4.0	33.2 ± 6.2	<0.001
BFP at 10–14 weeks, %, Mean ± SD	28.9 ± 4.4	28.5 ± 4.0	34.1 ± 6.1	<0.001
estimated BFP change rate, %, Median (IQR)	0.0 (0.0, 0.1)	0.0 (0.0, 0.1)	0.0 (0.0, 0.1)	0.477
estimated BFP change rate (10–14w), ‰, Median (IQR)	0.4 (−0.2, 0.9)	0.4 (−0.2, 0.9)	0.4 (0.0, 1.0)	0.477

Data are mean ± SD or median (IQR). P values: t-test, Mann-Whitney U, or chi-square. BFP, body fat percentage; BMI, body mass index; AST, aspartate aminotransferase; ALT, alanine aminotransferase.

### 3.2 Dose-response associations and thresholds

**Figure 2:**
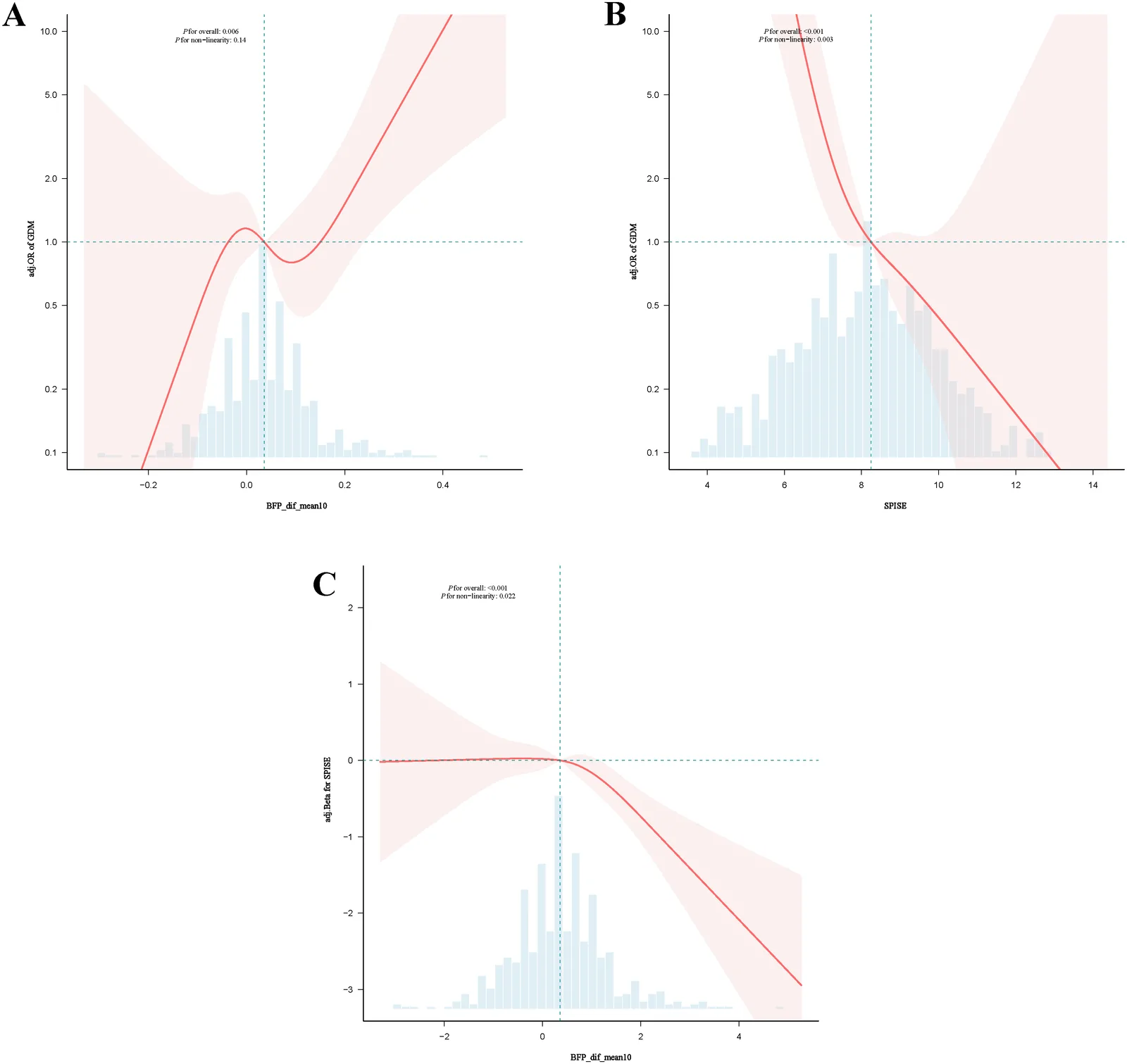
Three-panel restricted cubic spline plots showing adjusted associations among estimated BFP change rate, SPISE, and GDM. Panel **(A)** displays the adjusted odds ratio for GDM across estimated BFP change rate. Panel **(B)** displays the adjusted odds ratio for GDM across SPISE. Panel **(C)** displays the adjusted regression coefficient for continuous SPISE across estimated BFP change rate. Red curves represent adjusted estimates, pink shaded areas represent 95% confidence intervals, and light-blue histograms show exposure distributions. Horizontal dashed lines indicate an odds ratio of 1 in panels **(A)** and **(B)** and a regression coefficient of 0 in panel **(C)**.

Restricted cubic splines indicated that higher estimated BFP change rate was linked to greater GDM risk (P overall=0.006), without statistically significant evidence of non-linearity (P non-linearity=0.140) (**Figure 2A**). SPISE showed a non-linear inverse relationship with GDM risk (P overall < 0.001; P non-linearity = 0.003) (**Figure 2B**). Segmented logistic regression estimated the SPISE breakpoint at 7.089 (95% CI 7.046-7.132). For SPISE values below 7.089, the adjusted OR per one-unit higher SPISE was 0.127 (95% CI 0.049-0.328; P < 0.001). For values at or above 7.089, the corresponding slope was attenuated and not significant (OR 0.616, 95% CI 0.237-1.600; P = 0.320).The likelihood ratio test favored the two-piecewise model (P < 0.001) (**Table 2**).

**Table 2:** Segmented regression analysis for the association between SPISE and GDM risk with threshold effect.

Item	N	Breakpoint OR (95%CI)	*P* value
Breakpoint estimate	586	7.089 (7.046–7.132)	—
SPISE < 7.089 - segment effect 1	160	0.127 (0.049–0.328)	<0.001
SPISE ≥ 7.089 - segment effect 2	426	0.616 (0.237–1.600)	0.320
Likelihood ratio test	—	—	<0.001

The breakpoint was identified by two-piecewise logistic regression. Effect sizes are odds ratios (95% CI). The likelihood ratio test compares the piecewise model against the linear model. OR, odds ratio; CI, confidence interval.

Both full-cohort logistic RCS models and the SPISE-GDM segmented model included all 586 participants and 37 GDM events. The estimated BFP change rate-GDM model contained six non-intercept parameters, corresponding to 6.2 events per parameter, whereas the SPISE-GDM model contained seven non-intercept parameters, corresponding to 5.3 events per parameter. When the estimated breakpoint was counted as a model parameter, the segmented SPISE-GDM analysis likewise involved seven non-intercept parameters, corresponding to 5.3 events per parameter. Diagnostic findings for the estimated BFP change rate-GDM and SPISE-GDM RCS models are presented in **Supplementary Figures 3**, **S4**, respectively. All VIFs were below 5; values were close to 1 in the estimated BFP change rate-GDM model, while values were approximately 3.5 for SPISE and prepregnancy BMI and close to 1 for maternal age, parity, and gestational week at testing in the SPISE-GDM model. No observation exceeded the diagnostic influence contour boundaries in either model, providing no evidence that a single observation dominated the fitted associations. These diagnostic findings do not establish model stability or exclude small-sample overfitting and support cautious interpretation.

estimated BFP change rate also showed a non-linear inverse association with SPISE (P overall < 0.001; P non-linearity = 0.022) (**Figure 2C**). Piecewise linear regression placed the estimated BFP change rate breakpoint at 1.196. The inverse association was significant on both sides of the breakpoint, with a sharper decline in SPISE above the breakpoint (**Supplementary Table 2**).

### 3.3 Stratified risk visualization by SPISE threshold

**Figure 3:**
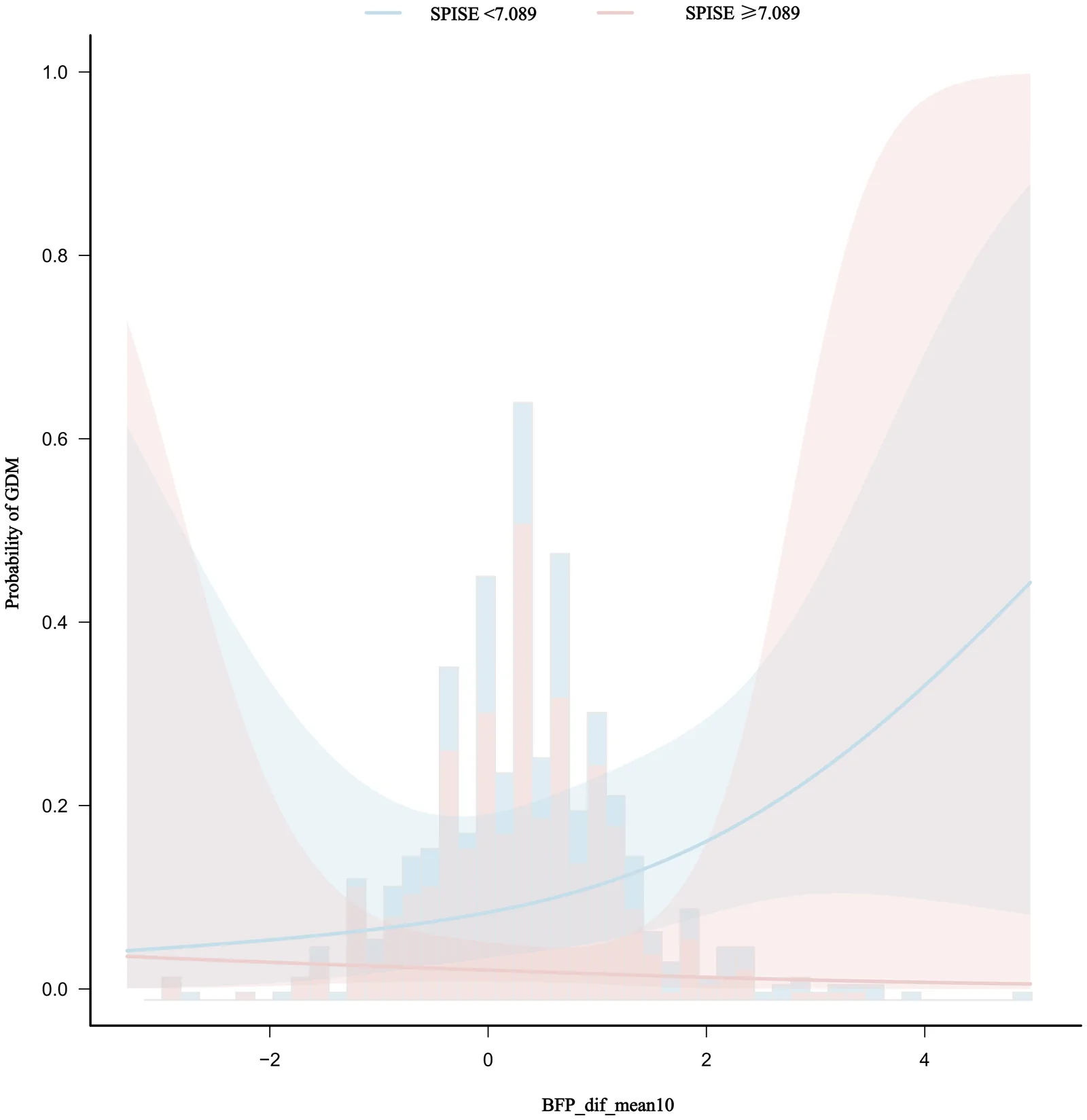
Line plot showing the adjusted predicted probability of GDM across estimated BFP change rate, stratified by SPISE category. The blue curve represents participants with SPISE below 7.089, and the red curve represents those with SPISE of 7.089 or higher. Shaded areas indicate 95% confidence intervals. The predicted probability increases with estimated BFP change rate in the lower-SPISE group but remains low in the higher-SPISE group. Overlaid histograms show the distribution of estimated BFP change rate within each SPISE group.

Spline visualization stratified by the SPISE threshold suggested that the association between estimated BFP change rate and GDM was concentrated mainly in the lower-SPISE stratum. The higher-SPISE stratum showed a flatter risk curve (**Figure 3**). This pattern is consistent with SPISE as a metabolic context in which early estimated BFP gain is more closely tied to GDM risk.

### 3.4 Mediation analysis

**Figure 4:**
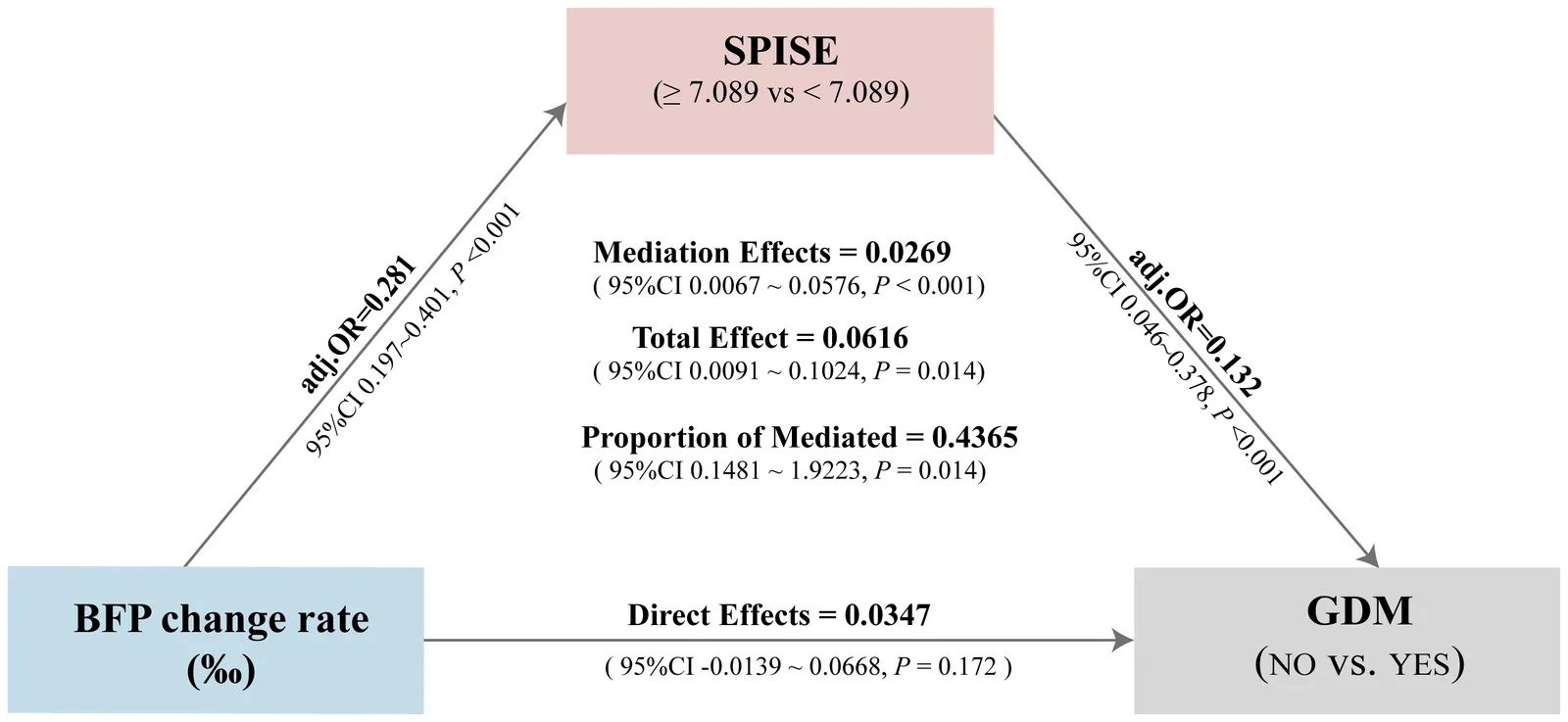
Path diagram illustrating the exploratory mediation analysis of estimated BFP change rate, SPISE, and GDM. Estimated BFP change rate is shown as the exposure, SPISE dichotomized at 7.089 as the mediator, and GDM as the outcome. Arrows indicate the exposure-to-mediator, mediator-to-outcome, and direct exposure-to-outcome paths. Adjusted odds ratios with confidence intervals and P values are displayed for the fitted paths. The center of the diagram reports the estimated indirect, direct, and total effects and the proportion mediated.

In the model using dichotomized SPISE as the mediator, higher estimated BFP change rate was linked to a lower probability of belonging to the higher-SPISE group (adjusted OR 0.281, 95% CI 0.197-0.401; P < 0.001). Higher SPISE was linked to lower GDM odds (adjusted OR 0.132, 95% CI 0.046-0.378; P < 0.001). The indirect effect through SPISE was significant (estimate 0.0269, 95% CI 0.0067-0.0576; P < 0.001), while the direct effect was not significant (estimate 0.0347, 95% CI -0.0139 to 0.0668; P = 0.172). The estimated mediated proportion was 43.65% (P = 0.014) (**Figure 4**).

### 3.5 Sensitivity analyses

The mediation results were similar when SPISE was treated as a continuous mediator. estimated BFP change rate was inversely linked to continuous SPISE (adjusted beta -0.443, 95% CI -0.510 to -0.377; P < 0.001), and higher SPISE was linked to lower odds of GDM (adjusted OR 0.296, 95% CI 0.181-0.484; P < 0.001). The indirect effect remained significant (estimate 0.0254, 95% CI 0.0113-0.0396; P = 0.0020). The mediated proportion was greater than 1 because the direct effect was close to zero and not significant. This value should be interpreted as evidence that SPISE largely accounted for the estimated BFP change rate-GDM association, not as a literal percentage (**Supplementary Figure 1**).

In the propensity score matched analysis, the matched set included 124 women, with 62 women in each SPISE group. Balance improved after matching for age, parity, pre-pregnancy BMI, and gestational week at testing (**Supplementary Figure 2**). In available-case outcome analysis after matching, GDM occurred in 9 of 62 women (14.5%) with SPISE < 7.089 and in 0 of 62 women (0.0%) with SPISE ≥7.089 (P = 0.003). Other available obstetric outcomes did not differ significantly between groups (**Table 3**).

**Table 3:** Available clinical outcomes in the PSM-matched cohort stratified by SPISE threshold.

Variable	Total (n = 124)	SPISE < 7.089 (n = 62)	SPISE ≥7.089 (n = 62)	*P* value
GDM, n (%)	9 (7.3)	9 (14.5)	0 (0)	0.003
PTB, n (%)	4 (3.2)	3 (4.8)	1 (1.6)	0.619
HDP, n (%)	3 (2.5)	1 (1.6)	2 (3.4)	0.612
Gestational week at delivery, Mean ± SD	39.1 ± 1.2	38.9 ± 1.1	39.3 ± 1.2	0.099
Cesarean delivery, n (%)	51 (41.1)	29 (46.8)	22 (35.5)	0.201
Birth weight (kg), Mean ± SD	3.3 ± 0.4	3.3 ± 0.3	3.3 ± 0.4	0.635
LGA, n (%)	14 (11.3)	9 (14.5)	5 (8.1)	0.256

Data are mean ± SD or n (%). GDM, gestational diabetes mellitus; PTB, preterm birth; HDP, hypertensive disorders of pregnancy; LGA, large for gestational age. GDM was compared using Fisher’s exact test because the higher-SPISE group had zero events and expected cell counts were small.

In the third sensitivity analysis, one participant who developed GDM was excluded because fasting glucose was missing. Among the remaining 585 participants (36 GDM events), ln(HOMA-IR) was positively associated with subsequent GDM (P overall = 0.002), with no evidence of departure from linearity (P non-linearity = 0.823) (**Supplementary Figure 6**). The model contained seven non-intercept parameters (5.1 events per parameter). Because higher HOMA-IR indicates greater insulin resistance, the direction of this association was consistent with the inverse SPISE-GDM association.

## 4 Discussion

### 4.1 Principal findings

This exploratory secondary analysis produced three main findings. First, faster estimated BFP change from before pregnancy to 10–14 weeks was linked to higher GDM risk. Second, first-trimester SPISE showed a threshold-dependent inverse relationship with GDM, with the threshold estimated near 7.089. Third, SPISE accounted statistically for part of the association between estimated BFP change rate and later GDM in both dichotomized and continuous mediation analyses.

### 4.2 Interpretation in the context of existing evidence

These findings fit current models of GDM biology. GDM occurs when insulin secretion does not adequately compensate for the insulin resistance of pregnancy (1, 8, 9). Maternal adiposity and dyslipidemia add to this metabolic burden, and a triglyceride-rich, low-HDL pattern is closely related to insulin resistance (9, 14). SPISE combines BMI, triglycerides, and HDL-C into a fasting index validated against clamp-derived insulin sensitivity (14). In this analysis, the inverse association between SPISE and GDM is consistent with SPISE serving as an early marker of impaired insulin sensitivity.

This analysis extends previous work in two ways. It focuses on adiposity change, moving beyond analyses based only on static adiposity. Women who developed GDM had higher pre-pregnancy and early-pregnancy BFP, and the adjusted spline results suggest that the early rate of change may add risk information. SPISE also appeared to capture part of the metabolic meaning of BFP change. Clinically, BFP change may reflect a rising adiposity burden, while SPISE may indicate whether that burden is accompanied by an insulin-resistant lipid profile.

### 4.3 mTOR-mediated nutrient sensing and metabolic homeostasis

In addition to the lipid and anthropometric inputs incorporated in SPISE, mechanistic target of rapamycin (mTOR) signaling provides a broader framework for nutrient sensing and metabolic homeostasis. mTOR complex 1 (mTORC1) integrates amino acid, energy, and insulin/growth-factor signals and regulates anabolic metabolism, autophagy, and cellular adaptation to nutrient availability; together with insulin/IGF-1 and AMPK signaling, it helps coordinate metabolic homeostasis (31). In pancreatic beta-cell models, the amino acid-Rab1A-mTORC1-PDX1 axis has been linked to beta-cell maintenance and insulin expression, while mTORC1-mediated phosphorylation of PDX1 at serine 61 influences its stability and transcriptional activity (32, 33). These experimental findings provide a possible biological context for relationships among nutrient sensing, insulin sensitivity, and GDM, but they do not demonstrate mTOR involvement in our results. The available dataset contained no measures of amino acid availability, mTOR activity, or downstream phosphorylation markers. Thus, this interpretation remains speculative and requires prospective pregnancy studies integrating mTOR-related measurements with direct assessments of body composition and insulin sensitivity.

### 4.4 Threshold and mediation findings

The relationship between SPISE and GDM was not constant across the observed range. Risk separation was concentrated below the threshold, where increases in SPISE were strongly associated with lower GDM odds. Above the threshold, additional SPISE increases were not linked to a statistically significant reduction in risk. This pattern suggests a possible saturation effect: when insulin sensitivity is relatively preserved, other determinants of GDM may become more influential. In the low-SPISE range, even modest differences in insulin sensitivity may matter clinically.

The mediation findings should be interpreted as statistical mediation rather than proof of causality. The timing of measurement supports the proposed sequence, because BFP change and SPISE were assessed early in pregnancy and GDM was diagnosed later. Observational mediation still relies on assumptions that cannot be fully tested, including the absence of unmeasured exposure-mediator, mediator-outcome, and exposure-outcome confounding (25, 26). In the continuous-SPISE model, the mediated proportion exceeded 1 because the direct effect was near zero and unstable in a small sample. The direction and statistical significance of the indirect effect are more informative than the exact proportion.

### 4.5 Clinical implications

If validated externally, combining early-pregnancy BFP change with SPISE could help stratify metabolic risk before routine GDM screening (6, 7). Women with rapid BFP change and low SPISE may be candidates for earlier nutrition counseling, physical activity guidance, and closer glucose monitoring (3, 7). The current SPISE threshold should not yet be used as a clinical cut-point. It was derived from one dataset, with a small number of GDM events, and may vary across populations.

### 4.6 Strengths

This study used a prospective dataset in which early-pregnancy metabolic measures were collected before GDM assessment. The analysis tested non-linear dose-response relationships without imposing a linear pattern (20, 21). Mediation analyses, sensitivity checks, and PSM examined a coherent metabolic pathway and the potential clinical usefulness of the SPISE threshold (24–28).

### 4.7 Limitations

Several limitations should be considered. First, this was a *post hoc* secondary analysis, and the findings should therefore be regarded as hypothesis-generating. Second, only 37 GDM events were available, which may have limited model stability and the precision of the effect estimates (29). Although the breakpoint analysis retained all 586 participants across the full observed SPISE range, the breakpoint remained data-derived and may be sample-dependent; it should not be regarded as a validated clinical cutoff. Third, BFP was estimated from available variables rather than measured directly using dual-energy X-ray absorptiometry, bioelectrical impedance, or skinfold assessment (12). The publicly available dataset contained pre-pregnancy BMI but did not provide BMI at 10–14 weeks as a separate variable or the corresponding height and weight measurements. BMI at 10–14 weeks was therefore algebraically recovered from the BMI component embedded in HSI. Although this approach recovers rather than predicts BMI from the HSI formula, rounding, limited numerical precision, or undocumented processing of the publicly released variables may have introduced measurement error. Because directly measured BMI at 10–14 weeks was unavailable, we could not independently validate the reconstructed value or directly assess whether the reconstruction materially affected the findings. Consequently, results involving reconstructed BMI, estimated BFP change, and SPISE should be interpreted cautiously. Future prospective studies should use directly measured first-trimester anthropometric and body-composition data and formally evaluate the robustness of these findings to BMI reconstruction. Finally, residual confounding by unmeasured factors, including diet, physical activity, family history of diabetes, gestational weight-gain patterns, socioeconomic factors, and genetic susceptibility, cannot be excluded (13, 15).

The ln(HOMA-IR) sensitivity analysis was *post hoc* and relied on a single fasting glucose and insulin measurement. HOMA-IR is a surrogate that primarily reflects fasting or hepatic insulin resistance and may be affected by insulin-assay variability; it is not equivalent to clamp-derived insulin sensitivity. Accordingly, the concordant result should be viewed as supportive sensitivity evidence rather than independent validation.

With respect to model complexity, the four-knot estimated BFP change rate-GDM logistic RCS model contained six non-intercept parameters for 37 events (6.2 events per parameter), whereas the four-knot SPISE-GDM logistic RCS model contained seven non-intercept parameters (5.3 events per parameter). Vittinghoff and McCulloch showed that the conventional 10-events-per-variable rule may be relaxed under some circumstances (30); In both logistic RCS models, no substantial multicollinearity or single highly influential observation was identified; nevertheless, model instability and overfitting due to the small number of events cannot be entirely excluded.

## 5 Conclusion

Faster early-pregnancy increase in estimated BFP was linked to higher later GDM risk. Lower first-trimester SPISE showed a threshold-like inverse association with GDM and statistically yielded an indirect-effect estimate involving SPISE. These findings support the hypothesis that early estimated BFP gain was associated with a higher risk of subsequent GDM through an insulin-resistant phenotype reflected by SPISE. Larger multiethnic cohorts with directly measured body composition are needed before clinical translation.

## Data Availability

Publicly available datasets were analyzed in this study. This data can be found here: https://doi.org/10.1371/journal.pone.0221400.

## Supplementary material

Supplementary Figure 1 Exploratory mediation analysis using continuous SPISE. The exposure was the estimated BFP change rate, the mediator was continuous SPISE, and the outcome was GDM. Arrows show the fitted exposure-mediator and mediator-outcome paths; the displayed quantities summarize the indirect, direct, and total effects and the proportion mediated. Confidence intervals were obtained from 1, 000 bootstrap resamples, with adjustment for maternal age, parity, and prepregnancy BMI. Because the direct effect was close to zero, a proportion mediated greater than 100% can occur and should not be interpreted as a literal percentage of a causal effect. Abbreviations: BFP, body fat percentage; BMI, body mass index; GDM, gestational diabetes mellitus; SPISE, single-point insulin sensitivity estimator.

Supplementary Figure 2 Covariate balance before and after 1:1 propensity-score matching for low versus high SPISE. Standardized mean differences are shown for maternal age, parity, prepregnancy BMI, and gestational week at testing in the unmatched cohort and in the matched sample of 62 participants per group. The vertical reference at an absolute standardized mean difference of 0.10 denotes the prespecified criterion for acceptable balance. This diagnostic evaluates balance in measured covariates and does not exclude residual confounding by unmeasured factors. Abbreviations: BMI, body mass index; SPISE, single-point insulin sensitivity estimator.

Supplementary Figure 3 Diagnostic plots for the four-knot logistic RCS model relating the estimated BFP change rate to GDM (N = 586; 37 GDM events; six non-intercept parameters; 6.2 events per parameter). Panel A compares observed counts for GDM = 0 and GDM = 1 with aggregate model-predicted counts and their uncertainty intervals. Panel B plots standardized residuals against leverage (hii); dashed contours indicate the influence boundaries, and labels identify observations with comparatively high leverage or residuals. Panel C displays variance inflation factors on a logarithmic scale; values below 5 were interpreted as indicating no substantial multicollinearity. The model was adjusted for maternal age, parity, and prepregnancy BMI. These diagnostics assess apparent fit, influence, and collinearity in the analyzed sample but do not constitute external validation or eliminate the possibility of small-sample overfitting. Abbreviations: BFP, body fat percentage; BMI, body mass index; GDM, gestational diabetes mellitus; RCS, restricted cubic spline.

Supplementary Figure 4 Diagnostic plots for the four-knot logistic RCS model relating SPISE to GDM (N = 586; 37 GDM events; seven non-intercept parameters; 5.3 events per parameter). Panel A compares observed counts for GDM = 0 and GDM = 1 with aggregate model-predicted counts and their uncertainty intervals. Panel B plots standardized residuals against leverage (hii); dashed contours indicate the influence boundaries, and labels identify observations with comparatively high leverage or residuals. Panel C displays variance inflation factors on a logarithmic scale; values below 5 were interpreted as indicating no substantial multicollinearity. The model was adjusted for maternal age, parity, prepregnancy BMI, and gestational week at testing. These diagnostics assess apparent fit, influence, and collinearity in the analyzed sample but do not constitute external validation or eliminate the possibility of small-sample overfitting. Abbreviations: BMI, body mass index; GDM, gestational diabetes mellitus; RCS, restricted cubic spline; SPISE, single-point insulin sensitivity estimator.

Supplementary Figure 5 Distributions of HOMA-IR and ln(HOMA-IR) among participants with complete HOMA-IR data (N = 585). Panel A shows untransformed HOMA-IR, and panel B shows its natural logarithm, ln(HOMA-IR). In each panel, the upper display presents ordered individual observations together with a horizontal boxplot, and the lower display presents a histogram with an overlaid kernel-density curve. The box indicates the interquartile range, the internal line indicates the median, whiskers extend to the non-outlying range, and individual circles denote outlying observations. The logarithmic transformation was used to reduce the marked right skewness of untransformed HOMA-IR before regression modeling. HOMA-IR was calculated as fasting insulin (μU/mL) × fasting glucose (mg/dL)/405. Abbreviation: HOMA-IR, homeostasis model assessment of insulin resistance.

Supplementary Figure 6 Adjusted restricted cubic spline association between ln(HOMA-IR) and GDM in the auxiliary sensitivity analysis (N = 585; 36 GDM events; seven non-intercept parameters; 5.1 events per parameter). Four knots were placed at the 5th, 35th, 65th, and 95th percentiles of ln(HOMA-IR). The solid curve shows the adjusted OR for GDM, the shaded band shows the 95% confidence interval, and the histogram shows the ln(HOMA-IR) distribution. The horizontal line denotes OR = 1, and the vertical line denotes the reference value of 0.4323. The model was adjusted for maternal age, parity, prepregnancy BMI, and gestational week at testing. The Wald tests yielded P overall = 0.002 and P non-linearity = 0.823, indicating an overall association without evidence of departure from linearity. One participant who developed GDM lacked fasting glucose and was excluded from this complete-case sensitivity analysis. Because this was a *post hoc* analysis with limited events, it should be interpreted as exploratory rather than as evidence of predictive performance or causality. Abbreviations: BMI, body mass index; GDM, gestational diabetes mellitus; HOMA-IR, homeostasis model assessment of insulin resistance; OR, odds ratio.
