# Supplementary material for: First-trimester SPISE mediates the association between early-pregnancy body fat percentage change and gestational diabetes mellitus: an exploratory secondary analysis of a prospective cohort
Source: Front Endocrinol (Lausanne). 2026 Sep 9;17:1931823. doi: 10.3389/fendo.2026.1931823 (PMC13597346; doi:10.3389/fendo.2026.1931823)
Supplement: Supplementary file 1 [file Table1.docx]

# Supplementary Material

**Supplementary Table S1. Comparison of basic characteristics between included and excluded populations.**

| Variable | Total (n = 623) | Excluded population (n = 37) | Included population (n = 586) | *P* value |
| --- | --- | --- | --- | --- |
| GDM, n (%) | 39 (6.4) | 2 (8.3) | 37 (6.3) | 0.661 |
| Age, Mean ± SD | 32.1 ± 3.9 | 32.2 ± 5.3 | 32.1 ± 3.8 | 0.812 |
| Parity, n (%) | 299 (48.0) | 20 (54.1) | 279 (47.6) | 0.447 |
| Pre-pregnancy BMI, Mean ± SD | 22.1 ± 3.5 | 23.0 ± 3.5 | 22.0 ± 3.5 | 0.120 |
| Gestational week at testing, Mean ± SD | 12.4 ± 0.5 | 12.5 ± 0.4 | 12.4 ± 0.5 | 0.443 |
| AST, IU/L, Mean ± SD | 17.8 ± 8.1 | 18.7 ± 6.6 | 17.8 ± 8.1 | 0.690 |
| ALT, IU/L, Median (IQR) | 11.0 (8.0, 15.0) | 13.0 (9.5, 17.5) | 11.0 (8.0, 15.0) | 0.303 |
| TG, mg/dL, Mean ± SD | 119.5 ± 48.2 | 141.1 ± 64.5 | 118.8 ± 47.6 | 0.060 |
| HDL, mg/dL, Mean ± SD | 65.0 ± 13.6 | 64.6 ± 15.6 | 65.0 ± 13.5 | 0.909 |

Note. Data are mean ± SD, median (IQR), or n (%). P values: t-test, Mann-Whitney U, or chi-square/Fisher. Abbreviations: GDM, gestational diabetes mellitus; BMI, body mass index; AST, aspartate aminotransferase; ALT, alanine aminotransferase; TG, triglycerides; HDL, high-density lipoprotein; BFP, body fat percentage.

**Table S2. Threshold effect analysis of BFP_dif10 on SPISE using two-piecewise linear regression.**

| Item | N | β / Breakpoint | Lower 95% CI | Upper 95% CI | *P* value |
| --- | --- | --- | --- | --- | --- |
| Breakpoint estimate | 583 | 1.196 | 1.155 | 1.237 | — |
| Below breakpoint (< 1.196) | 503 | −0.370 | −0.467 | −0.273 | <0.001 |
| Above breakpoint (≥ 1.196) | 80 | −0.677 | −0.983 | −0.371 | <0.001 |
| Likelihood ratio test | — | — | — | — | 0.022 |

Note. The breakpoint was identified by two-piecewise linear regression. Effect sizes are beta coefficients (95% CI). The likelihood ratio test compares the piecewise model against the linear model. Abbreviations: CI, confidence interval.
